# Exercise therapy to improve mobility, active behaviour and quality of life of chronic kidney disease patients with peripheral artery disease: study protocol for the EXACT-CKDPAD multicentre randomised controlled trial

**DOI:** 10.1136/bmjsem-2025-002740

**Published:** 2025-07-15

**Authors:** Fabio Manfredini, Vincenzo Panuccio, Yuri Battaglia, Alda Storari, Nicola Lamberti, Giovanni Piva, Marco Veronesi, Rocco Tripepi, Natascia Rinaldo, Anna Crepaldi, Claudia Momentè, Angela Piccinini, Luca Traina, Aaron Thomas Fargion, Sofia Straudi, Andrea Baroni, Alfredo De Giorgi, Carlotta Martinuzzi, Marcello Monesi, Alessandro Capitanini, Filippo Aucella, Adamasco Cupisti, Francesca Mallamaci, Carmine Zoccali, Roberto Manfredini

**Affiliations:** 1 Department of Neuroscience and Rehabilitation, University of Ferrara, Ferrara, Italy; 2 Unit of Vascular Rehabiltiation and Exercise Medicine, University Hospital of Ferrara, Ferrara, Italy; 3 Grande Ospedale Metropolitano, Reggio Calabria, Italy; 4 Department of Medicine, University of Verona, Verona, Italy; 5 Pederzoli Hospital, Nephrology and Dialysis Unit, Peschiera del Garda, Italy; 6 Unit of Nephrology, University Hospital of Ferrara, Ferrara, Italy; 7 Institute of Clinical Physiology, National Research Council CNR-IFC, Reggio Calabria, Italy; 8 Unit of Vascular and Endovascular Surgery, University Hospital of Ferrara, Ferrara, Italy; 9 Unit of Physical and Rehabilitation Medicine, University Hospital of Ferrara, Ferrara, Italy; 10 Clinica Medica Unit, University Hospital of Ferrara, Ferrara, Italy; 11 Primary Care Department, Unit of Diabetology, University Hospital of Ferrara, Ferrara, Italy; 12 Nephrology and Dialysis Unit, San Jacopo Hospital, Pistoia, Italy; 13 Nephrology and Dialysis Unit, San Giovanni Rotondo Foggia, Italy; 14 Department of Clinical and Experimental Medicine, University of Pisa, Pisa, Italy; 15 Clinical Epidemiology of Renal Diseases and Hypertension Unit, Institute of Clinical Physiology, Reggio Calabria, Italy; 16 Institute of Molecular Biology and Genetics (Biogem), Ariano Irpino, Italy; 17 Renal Research Institute, New York, New York, USA; 18 Department of Medical Sciences, University of Ferrara, Ferrara, Italy

**Keywords:** Exercise, Walking, Kidney, Cardiovascular

## Abstract

The combination of chronic kidney disease (CKD) and peripheral artery disease (PAD) enhances the already present high cardiovascular risk, exposing the affected patients to unfavourable long-term clinical outcomes. Physical exercise is considered an effective treatment for reducing sedentary behaviour and improving quality of life, but several barriers limit patient participation. In this parallel-design, single-blinded, randomised controlled trial, we will enrol 130 patients with concomitant CKD at stages III and IV and PAD at the claudication stage to be randomised into a 6-month exercise (Ex) or control (Co) intervention. The Ex programme will consist of two daily 10 min interval walking sessions (1 min of walking followed by 1 min of resting), with gait speed controlled via a metronome and increased approximately weekly. The Co group will receive standard nephrological care. Outcomes will be assessed before and after treatment, as well as at the 12-month follow-up. The primary outcome will be the 6 min walking distance. The secondary outcomes will include quality of life, lower limb and handgrip strength, body composition and bone mineral density, as well as circulating indexes of kidney function and long-term clinical outcomes. Since no trials have been published that purposely enrol this high-risk population (CKD-PAD), the eventual positive results will validate a simple, pain-free exercise intervention that can be carried out at home to improve patients’ mobility and quality of life. Trial registration number: NCT06621264.

**WHAT IS ALREADY KNOWN ON THIS TOPIC**

**WHAT THIS STUDY ADDS**

- The EXACT-CKDPAD study aims to investigate whether a simple, pain-free, home-based walking programme is feasible and effective in improving patients’ mobility, quality of life and long-term clinical outcomes.

**HOW THIS STUDY MIGHT AFFECT RESEARCH, PRACTICE OR POLICY**

- If the trial results confirm the initial hypothesis, a pain-free, simple walking programme will be made available to a wide number of patients worldwide, allowing them to carry it out at home, thereby improving their physical functioning and potentially reducing the burden on national healthcare services.

## Introduction

 Chronic kidney disease (CKD) is one of the fastest-growing non-communicable diseases. It is responsible for 1.2 million deaths per year and is projected to become the fifth leading cause of years of life lost worldwide by 2040.^1^,^3^ This high risk of morbidity and mortality^2^ is the sum of several critical factors affecting patients with CKD, who are exposed to an increased cardiovascular risk, including a higher risk of developing peripheral artery disease (PAD). In particular, PAD is related to adverse short-term and long-term clinical outcomes,^4 5^ considering the strong association between albuminuria and amputation.^6^

Approximately one out of four patients in the world with CKD is concomitantly affected by PAD.^7^ PAD prevalence in patients with reduced kidney function is significantly higher (24%) compared with subjects (3.7%) with normal kidney function (3.7%), and on the other hand, CKD prevalence is close to 40% in patients with PAD.^4^ These considerations delineate a subgroup of patients who share the same traditional risk factors (older age, diabetes, hypertension, hyperlipidaemia, smoking, physical inactivity) but also present unique risk factors, such as increased albuminuria, inflammation, oxidative stress, endothelial dysfunction and an unbalanced metabolic panel.^8 9^ These aspects are all closely linked in terms of both cause and effect to the sedentary attitude of the patient with CKD.^10^,^12^ Indeed, this high-risk population is poorly engaged in multidisciplinary secondary prevention programmes,^13 14^ and the need for effective interventions with sustainable benefits is reflected in the lack of previous randomised controlled trials (RCTs) focused on improving people’s mobility. In addition, reduced physical activity is associated with unfavourable outcomes in patients with CKD, including progressive deconditioning, poor control of risk factors, reduced muscle mass and fatigue up to sarcopenia.^12 15 16^ Exercise may counteract these effects in both CKD and PAD populations,^4 16^ and higher physical activity levels may contribute to better management of cardiovascular risk factors and, possibly, retard CKD progression.^17 18^ Indeed, several meta-analyses have been conducted in both populations, yielding promising results in terms of mobility, exercise capacity, quality of life, laboratory parameters and many other outcomes.^19^,^29^

However, despite the effectiveness of exercise and physical activity programmes, several barriers are still present for patients’ participation, both in CKD and PAD populations.^30 31^

To this end, the search for models of exercise prescription that are connectable with nephrology services should be implemented; however, no trials are available for the global management of this condition in the CKD-PAD population.^32^

In recent years, a home-based walking programme has been developed to overcome barriers to exercise among patients with CKD and PAD. It has been successfully tested with favourable effects on exercise capacity, physical functioning and quality of life in patients with CKD^33^,^35^ and PAD.^36^,^39^ We hypothesise that a very similar intervention could also be effective when both conditions are present.

Therefore, this multicentre RCT aims to test the effectiveness of a home-based pain-free exercise programme in a CKD-PAD population on mobility, quality of life, exercise capacity and long-term outcomes, including CKD progression, peripheral revascularisations and survival compared with usual care.

## Methods

### Study design and setting

This study protocol is reported following the Standard Protocol Items: Recommendations for Interventional Trials (SPIRIT) guidelines.^40^ The study is designed as a multicentre, parallel-group, single-blinded, RCT. The trial is available at Clinicaltrials.gov NCT06621264.

The study will take place at Sant’Anna University Hospital of Ferrara, which will act as the coordinating centre, as well as at Grande Ospedale Metropolitano Bianchi-Melacrino-Morelli of Reggio Calabria, Ospedale Pederzoli in Peschiera del Garda, the University of Verona and Casa Sollievo della Sofferenza in San Giovanni Rotondo. All units will cooperate in the enrolment of patients and data collection.

### Eligibility criteria

Patients will be included if the following criteria are respected: male and female patients aged >18 years affected by CKD at Kidney Disease Outcomes Quality Initiative (KDOQI) stages III or IV with concomitant PAD at Rutherford’s stages I–III; ability to walk independently; cognitive function to give informed consent identified by a Mini Mental Status Examination score≥20/30 and absence of clinical conditions contraindicating exercise therapy (eg, unstable angina, severe heart failure at New York Heart Association class IV, anaemia with Hb<100 g/L). Exclusion criteria are major amputations; major surgery planned in the next 3 months; known comorbid conditions that may limit survival to <2 years and inability or unwillingness to comply with protocol requirements.

**Figure 1:**
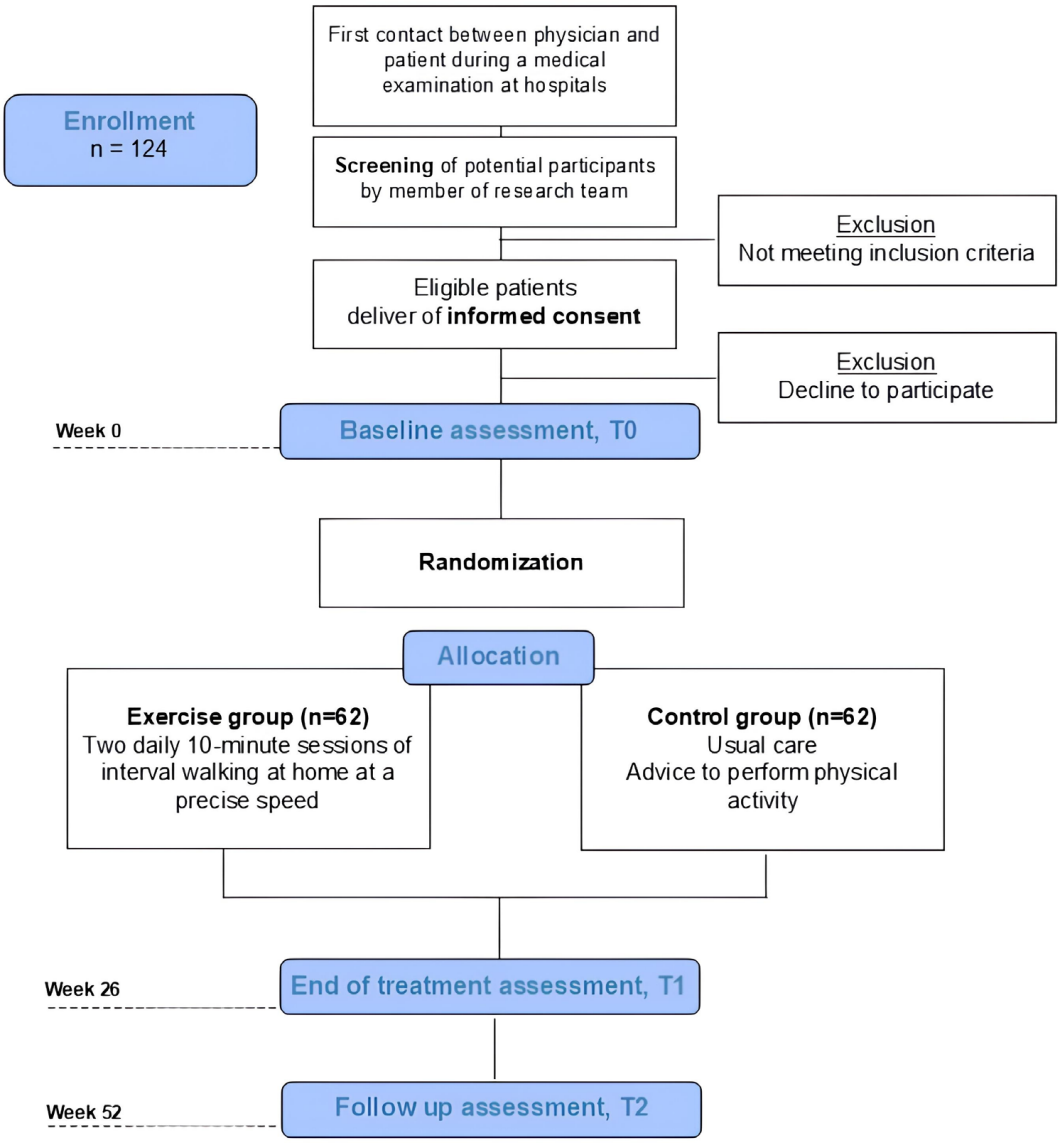
Flow chart of the study.

Each Operative Nephrology Unit database will select all potential patients. If the inclusion criteria are met, potential participants will be given a study information leaflet detailing the study’s objectives, procedures, time frame, risks and potential benefits, as well as the telephone contact details of the staff involved and the Consent Form (supplementary material). Within the following week, candidates will be contacted by telephone and asked about their decision. For those who decide to participate, an appointment will be scheduled at which signed informed consent will be requested, and a baseline assessment session will be performed. The total number of subjects screened will be recorded according to the Consolidated Standards of Reporting Trials guidelines (figure 1).

### Interventions

Patients randomised to both groups will receive optimisation of medical therapy and standard nephrological care. Follow-up visits and assessments for each group member will be performed by a specialised physician and an exercise physiologist

#### Experimental group

Patients enrolled in the experimental group will receive a detailed 6-month exercise prescription aimed at providing in-home, low-fatiguing walking training at light to moderate intensity (2–3 out of 10 on the CR10 Borg’s scale). The programme consists of two daily 10 min interval walking sessions (1 min of walking followed by 1 min of resting), which will be maintained at a constant pace throughout the entire programme. Walking speed will be prescribed according to the patient’s baseline walking capacity (approximately 50% lower than the habitual gait speed collected during the 6 min walking test). It will be increased weekly until reaching the habitual gait speed, following a specific scheme (table 1). Training intensity will be prescribed in step cadence and controlled by a digital metronome (https://www.youtube.com/watch?v=ki8YX_t-0jA) or a smartphone application that will lead the patient to walk at the right speed by listening to the prescribed walking pace (step/min). Each patient will be trained in the walking programme during the first visit. Training sessions will preferably be performed overground indoors at home to prevent the influence of weather or on a treadmill if patients prefer it.

**Table 1:** Weekly walking speed progression for the exercise group

Week	Speed (steps/min)
1	60
2	60
3	63
4	63
5	66
6	66
7	69
8	69
9	72
10	72
11	76
12	76
13	80
14	76
15	80
16	84
17	84
18	88
19	88
20	92
21	84
22	84
23	88
24	92
25	96
26	100

Each training session includes a 10 min interval training session with a walk:rest ratio of 1:1. The training speed is adjusted to ±3 steps/min according to the patient’s baseline capacity or other conditions that may limit walking speed.

Exercise prescriptions will be updated during two intermediate hospital visits in the first and third months. Patients will be provided with a digital application that has been properly developed to track pacing and record the correct training execution. During the intermediate visit, systolic and diastolic blood pressure will be monitored. A team member will conduct periodic, approximately weekly telephone surveys to encourage patients to complete their home exercise diary and report any problems they encounter during exercise, such as pain or discomfort so that the exercise prescription can be adjusted appropriately.

#### Control group

Patients enrolled in the control group will receive standard CKD-PAD care, including optimal medical therapy and nutritional advice.

### Concomitant care and recommendations

No restrictions of concomitant care physiotherapy or physical activity programmes are applied. Any participation in exercise programmes or changes in pharmacological therapy will be precisely recorded in both groups.

### Fidelity to treatment

Several actions will be taken to maintain a high level of adherence and interest among patients enrolled in the exercise group. First, the walking programme will be explained by the physician and tried by patients during the first visit to state the feasibility and friendliness of the programme. Second, patients will be encouraged to continue participating in the programme during follow-up visits in the first and third months and will receive fortnightly phone calls from a researcher responsible for monitoring patients’ health and addressing any possible doubts or concerns. Finally, next to an optimally written scheme and explanation of the programme, a properly designed training diary will be provided to each patient to record exercise execution and any potential symptoms. Patients in the control group will also receive a diary to record the physical activity performed for each day.

### Outcomes

Outcome measures will be collected at baseline (T0), after 6 months (T1), which corresponds to the end of the training programme for the experimental group, and at the 12-month (T2) follow-up by healthcare professionals blinded to patients’ allocation.

*The primary outcome* will be the measurement of the 6 min walking distance (6MWD) performed during the 6 min walking test.^41^ The variations in mobility assessed by 6MWD will be determined at the end of the programme in relation to the baseline. The patients will be instructed to walk back and forth along a 20 m corridor, aiming to cover as much distance as possible. Patients will also have to report the onset of any symptoms during the test, and the correspondent distance will be recorded as pain-free walking distance. When necessary, they will be allowed to stop as needed and restart whenever possible. Patients will also be monitored with a pulse oximeter to check their corresponding saturation. The rate of perceived exertion will be recorded on the 0–10 Borg scale at the end of the test.

*Secondary outcomes* will include the following:

- Lower limb perfusion. The haemodynamic severity of the disease will be assessed by the ankle-brachial index (ABI). This measure will be performed according to the published standards. The patient lies in a supine position, and blood pressure values at the ankle (from the posterior tibial and dorsalis pedis arteries) will be collected and compared with the systolic blood pressure. ABI is obtained by the ratio of the highest ankle value recorded and will be determined for both legs.^42^
- Foot perfusion and temperature will be measured using an infrared thermal camera (FlirOne Pro, Flir, Milan, Italy). Infrared thermography is a non-invasive technique which has not yet been employed systematically in clinical trials, used for easy and reliable foot perfusion detection with promising results in patients with PAD.^43^ A sample of five points covering the foot (anterior and posterior tibial, dorsalis pedis and plantar) will be measured and collected, as previously proposed in the literature.^44^
- The five-time sit-to-stand test will assess lower limb strength. Patients will rise from a standard-height chair with their arms folded across the chest five times as quickly as possible. The total time elapsed for completing the five repetitions is the score.^45^
- Handgrip strength will be measured using a standard dynamometer (Takei dynamometer). With one hand at a time, patients will be asked to perform three repetitions. The peak and mean values will be collected.
- Quality of life will be assessed by the Short-Form 36 questionnaire. This is a generic questionnaire that contains 36 questions referring to eight specific domains related to patient physical and mental health over the previous 4 weeks.^46^
- Bone mineral density will be measured through phalangeal quantitative ultrasound (QUS, DBM Sonic Bone Profiler, Igea s.r.l., Carpi) with an ultrasound signal of 1.25 MHz frequency and a methodology devoid of X-rays. QUS results are consistent with those obtained by standard DEXA.^47^ ;
- Body composition will be assessed with direct measures of height (cm), weight (kg) and waist circumference (cm), as well as lean body mass and lean body mass index through bioimpedance analysis (Akern, Italy).
- Circulating indexes of kidney function such as serum creatinine, estimated glomerular filtration rate (through the 2009 Chronic Kidney Disease Epidemiology Collaboration equation), albumin, urinary sediment and albuminuria-to-creatininuria ratio will be assessed by standard laboratory methods.
- Long-term clinical outcomes will also be recorded, including the number of patients requiring initiation of dialysis treatment within the 2-year follow-up period, as well as all-cause hospitalisation and mortality rates among the enrolled patients. In the event of positive findings, the number of days elapsed from enrolment to the event will be recorded for additional analysis, as well as the reasons and diagnoses. Long-term outcomes will be collected up to 3 years from the enrolment.

**Figure 2:**
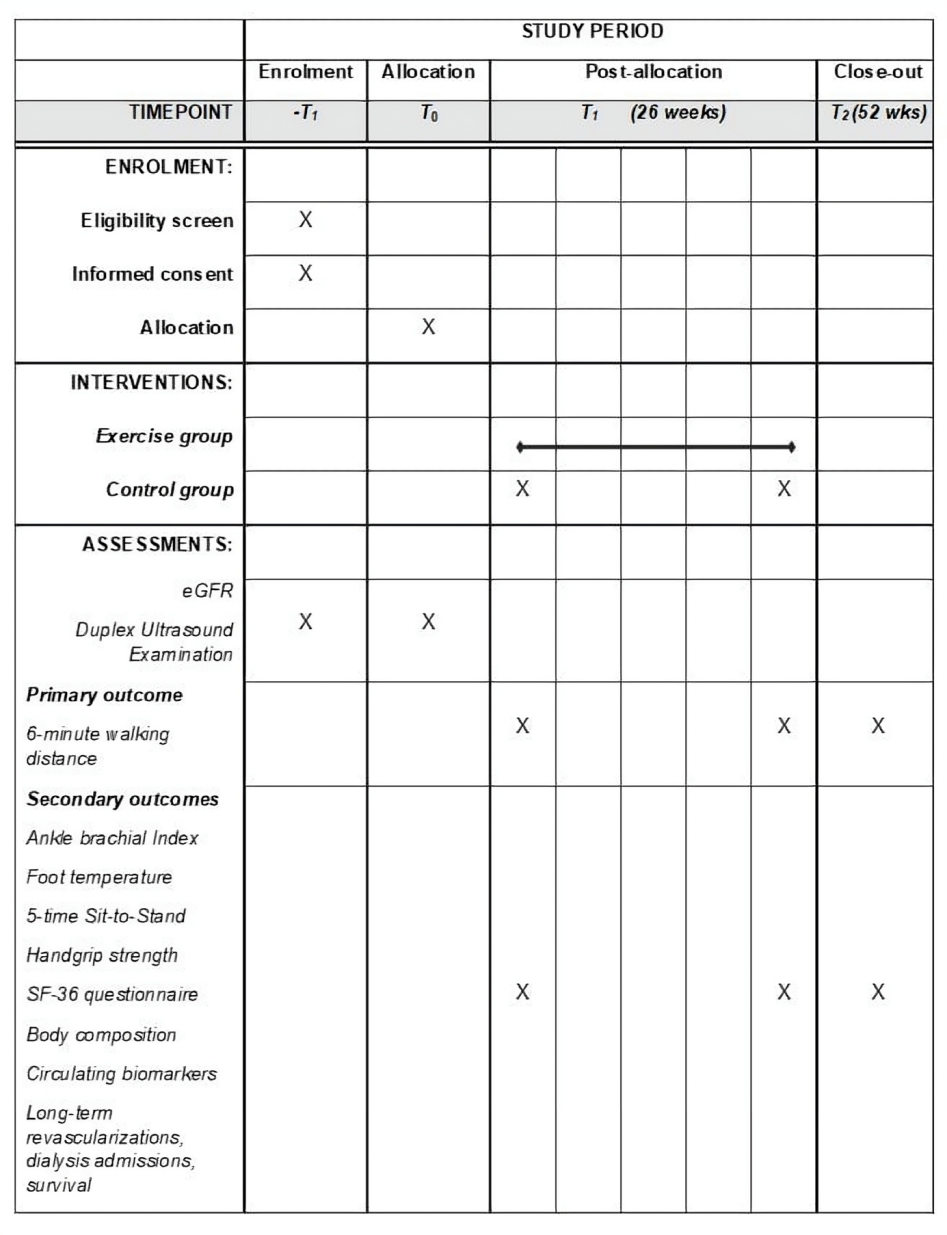
Study timeline according to Standard Protocol Items: Recommendations for Interventional Trials (SPIRIT) statement. eGFR, estimated glomerular filtration rate; SF-36, Short-Form 36.

Participants' timeline is reported in figure 2.

### Sample size and recruitment

The feasibility of this study is assessed by analysing the prevalence of patients with CKD-PAD in the hospitals involved in this project. We identified a cohort of over 200 patients potentially meeting the inclusion criteria. In each centre, a network of physicians, including nephrologists, internists and vascular surgeons, will be established to identify potentially eligible patients and facilitate enrolment. Every centre will identify a responsible person for enrolment to assess patients’ eligibility and manage their inclusion in the randomisation list.

We calculated the sample size, hypothesising the superiority of the experimental treatment over the control group. A sample of 96 patients, 48 in each group, is needed to detect a 30 m improvement in 6 min walking distance in favour of the experimental group, assuming an SD of improvement of 30 m, with a type I error of 0.05 and a power of 90%. To account for possible dropouts, the sample size will be increased by 30%, resulting in a total of 124 patients to be enrolled.

### Allocation

After the recruitment procedure at baseline, the physician responsible for enrolment in the Coordinating Centre will create the allocation sequence on a password-protected computer. An external administrator not involved in the trial will then randomise patients to one of the two groups using a computerised randomisation stratification approach to prevent selection bias. The random assignment will be communicated to investigators on the day of enrolment by accessing it on identification in an online repository. Only one coordinator of each centre will be permitted to access the participants’ allocation. The randomisation scheme (1:1 ratio) will be established using permuted blocks of random sizes. The block sizes will not be disclosed to ensure concealment. Finally, the subjects will be assigned to one of two study groups: the exercise (Ex) group or the control (Co) group.

### Blinding and data collection, management and analysis

Professionals in the management of the exercise programme will be responsible for outcome measurement, with one researcher assigned to each operational unit. An expert in data collection of body composition and bone mineral density will be responsible for collecting body composition parameters in every operating unit.

Two researchers blinded to the patients’ allocation will be responsible for entering the data into two electronic datasets, which will be compared with check for possible mistakes in data input. A password will protect every data set.

The researchers responsible for the statistical analyses will also be blinded to the patient group assignments. He will also be responsible for data management (quality control and data checking) and data analysis according to the different research hypotheses described.

Patient’s data will be treated following current regulations, including the 2016/679 European Union Act and D.Lgs. 30 June 2003, n. 196 s.m.i. Italian regulation, as well as any possible adjunctive measures deemed applicable by the Italian Data Protection Authority.

### Statistical methods

Standard methods for the analysis of RCTs will be employed. All analyses will be conducted using intention-to-treat, where any subject randomised to one arm remains in that arm regardless of whether they received the intervention.

Missing values, although we will make any effort possible to reduce their incidence, will be treated using the multiple imputation procedure. Moreover, a sensitivity analysis will be performed to assess the stability of the study’s conclusions, comparing the intention-to-treat analysis with an analysis that takes into account the level of participation in the intervention arm. A p value <0.05 will be considered statistically significant. Several statistical analysis software packages, including SPSS, MedCalc and Stata, will be used.

A Shapiro-Wilk test will verify the nature and distribution of the data. At baseline, the two groups will be compared in terms of demographics and primary and secondary outcome measures. Although our initial hypothesis suggested the superiority of the exercise group over the control group, to account for the possibility that participants in the control group might achieve better outcomes than those in the intervention arm, we will use two-tailed tests of significance for all analyses. For categorical variables, the χ^2^ test or Fisher’s test will be employed, as appropriate. Overtime between-group differences will be analysed using t-tests for symmetrically distributed data and analogous non-parametric tests, such as the Mann-Whitney test, for skewed data. A two-way repeated measures analysis of variance (factors: treatment, time) will be run to compare differences over time for secondary outcomes. Paired-sample t-tests or Wilcoxon tests will assess the within-group variations. Suppose a significantly different distribution between the two groups in the baseline outcome level is identified. In that case, a secondary analysis will be performed using multivariate modelling, such as analysis of covariance, to adjust for these factors. For the long-term outcomes, Kaplan-Meier curves with log-rank tests and Cox proportional hazard regression models will be performed.

### Harms

Potential risks, such as a major cardiac event or a fall, are expected to be rare. The intensity of the exercise proposed in this trial is low to moderate, interspersed with pauses and characterised by a low cardiovascular load. In our experience, among over 2000 patients at high cardiovascular risk with PAD, CKD, diabetes and/or stroke, programmes performing more than 400 000 home walking sessions for 6 months reported no falls or major events. Minor risks of participation, such as muscle soreness, fatigue or minor sprains or strains, are expected to occur in a small number of individuals during the training phase or assessment phase.

Anyway, all necessary procedures for the safety of the enrolled patients will be taken, including the exclusion of individuals with absolute contraindications to testing (inclusion/exclusion criteria) and careful monitoring of the testing phases by trained personnel (licensed and properly trained physical therapists/exercise physiologists). Direct supervision of trained operators will be employed during every phase of testing in which the participant is standing and/or walking. A medical clearance will be requested before initiating the exercise intervention.

Finally, proper insurance has been subscribed to cover all possible harms that may occur.

### Data monitoring, auditing and interim analyses

The study does not have a data monitoring committee. The research coordinator will conduct an interim analysis every 6 months to determine whether the study should be stopped, modified or continued. Any subsequent changes will be discussed by the research team and communicated to the funding agency and the Ethics Committee.

### Confidentiality

Participant confidentiality will be rigorously maintained throughout all phases of the study. Each participant will be assigned a unique study identification number, and all data will be de-identified before analysis. Personally identifiable information, including names and medical records, will be stored separately from study data in secure, access-restricted databases. Only authorised research personnel will have access to identifiable participant data, and all team members will receive training in confidentiality and data protection procedures. Electronic data will be stored on encrypted and password-protected systems, and any physical records will be secured in locked filing cabinets in restricted-access areas. Data will be used solely for the purposes outlined in the study protocol. No identifiable information will be disclosed in any publication, presentation or report resulting from this research.

### Dissemination

The results obtained from this RCT will be disseminated through multiple channels to ensure that the knowledge generated reaches relevant stakeholders, including the scientific community, healthcare professionals, policymakers and the general public. The primary method of dissemination will be through publication in peer-reviewed scientific journals relevant to the study’s field. Second, results will be presented at scientific conferences. They will be posted on clinical trial registries. Finally, a plain-language summary of the trial findings will be developed for study participants and the general public to be distributed through patient groups, hospital websites and the community.

Patients’ associations will be involved in the discussion of the results obtained after the analyses performed by the research team.

## Conclusions

This study will fill a knowledge gap in the literature as no trials have primarily focused on mobility in patients with concomitant CKD and PAD. This trial will propose a model of a home-based exercise program as a non-invasive alternative to manage the mobility impairment in this population. This type of model may reduce the rate of surgical or endovascular revascularisation and achieve better short-term and long-term clinical outcomes.

This study provides an example of the management of this population by care teams employing a collaborative, multidisciplinary approach, testing the feasibility of this approach in the nephrology departments of five different Italian regions and demonstrating its potential for wider application. In the event of positive results from this rigorously conducted trial, a simple, integrated model of exercise therapy, effective and low-burden for patients and families, will be validated.

The introduction of this model into clinical practice could be beneficial for managing CKD patients with PAD, reducing sedentariness, deconditioning and walking disability, and limiting related clinical and psychological consequences.

In addition, the attempt to slow the decline of renal function and prevent entry into dialysis treatment is a further sanitary, socioeconomic and environmental potential outcome of interest, particularly considering the potential application of this programme in different settings with limited resources. The validation of this feasible exercise programme may be a turning point for the health service, which could employ a cost-effective intervention strategy centred on patient empowerment.

## Data Availability

No data are available.
